# Different pitch configurations constrain the external and internal loads of young professional soccer players during transition games

**DOI:** 10.5114/biolsport.2023.124848

**Published:** 2023-04-07

**Authors:** Jose A. Asian-Clemente, Alberto Rabano-Muñoz, Luis Suarez-Arrones, Bernardo Requena

**Affiliations:** 1 Real Betis Balompié, Performance Department, Seville, Spain; 2 Department of Sport Sciences, Universidad Pablo de Olavide, Sevilla, Spain; 3 Football Science Institute, Granada, Spain; 4 FC Lugano, Performance Departament, Lugano, Switzerland

**Keywords:** Football, High-speed running, GPS, Time-motion, Physiological demands

## Abstract

The aims of this study were to compare the influence of transition game (TG) size on the external and internal loads of young professional soccer players and to describe the high-speed profile of these drills in response to pitch dimensions. Eighteen young professional soccer players (age: 16.1 ± 0.3 years; height: 178.3 ± 5.4 cm; weight: 70.1 ± 6.2 kg) performed a 3vs2 TG on pitches measuring 40 × 30 m (TG_30_), 40 × 50 m (TG_50_) and 40 × 70 m (TG_70_) m. Distance covered (DC); accelerations-decelerations above 1.0 m · s^−2^ and 2.5 m · s^−2^; rate of perceived exertion (RPE); maximal heart rate and time above 90%; DC at 18.0 to 21.0 km · h^−1^ (DC 18–20.9 km · h^−1^); DC at 21.0 to 23.9 km · h^−1^ (DC 21–23.9 km · h^−1^); DC above 24.0 km · h^−1^ (DC > 24 km · h^−1^); and peak speed and sprint profile (duration, distance and maximal speed) were measured. TG_30_ achieved lower DC, DC above 18 km · h^−1^, peak speed, sprint distance and RPE than TG_50_ and TG_70_ (p < 0.01 and p < 0.05) and lower sprint duration and maximal speed sprint than TG_70_ (p < 0.01). TG_30_ and TG_50_ achieved higher Acc > 1.0 and > 2.5 m · s^−2^ respectively than TG_70_ (p < 0.05). TG_70_ showed greater DC above 21 km · h^−1^, peak speed, sprint distance and maximal speed sprint than TG_50_ (p < 0.01). Soccer coaches should use larger TGs to overload variables related to high speed and sprint demands during training and smaller TG formats to stimulate the accelerations of the soccer players.

## INTRODUCTION

Currently training prescriptions follow a game-based approach in which ball-drill prescriptions are focused on the game as a whole [1]. The use of drill-based games such as small-sided games (SSGs) has become popular since they reflect the multidimensional stimulus provided by matches while allowing the coach to alter players’ specific responses by manipulating various task constraints [2–5]. Despite their popularity, it has been reported that these soccer tasks do not always reproduce the distances covered at high or very high speeds of the matches [6, 7]. Taking into account that during official matches counter-attacks are the actions in which players have to run at high or very high speeds [8] and that during the transitions the high-speed activities are 7–9-fold greater than the 90-min demands [9], coaches mostly choose transition games (TG) to develop these running skills in professional soccer through a specific game context. TGs are soccer tasks in which players continuously have to complete counter-attack situations using fast attacking and defensive transitions. Transitions or counter-attacks are crucial phases of the game since they have been found to be the most effective style of play for scoring goals, with more risks taking place during these moments [10, 11]. Despite the critical importance of the transitions in soccer, they have received scarce attention in the scientific literature. To date, only one study has investigated the physical demands of these actions during official matches, confirming that transitions exceeded the high-speed activities of the matches and nearly half of the game sprint distance [9]. Based on this, it is proposed that training programmes for professional soccer players should be focused on training high-speed running to improve sprint capacity with possession of the ball through transitional specific exercises [12], but only one study has analysed these drills during soccer training, showing that TGs induce more high-intensity requirements and lower variability in players’ responses than do small- and large-sided games [13]. By contrast, other training strategies such as supplementary time-efficient challenges in the form of high-speed straight runs, running involving directional changes, repeated sprints or modified-sided games have been widely studied [14]. For example, studies have demonstrated that introducing 30 s of running drills in a small-sided game, or changing zones during small-sided games, increases the distance covered by players at moderate and high speeds to a larger extent than the same tasks without these constraints [8, 15]. Considering this, coaches should develop TGs that stimulate the maximum physical outputs of the soccer players, and the manipulation of the pitch size during these drills needs to be examined [9]. Therefore, the aims of this study were (1) to compare the influence of the TG pitch size on external and internal loads in young professional soccer players and (2) to describe the high speed profile of these training drills with different pitch dimensions.

## MATERIALS AND METHODS

### Subjects

Eighteen young soccer players (age: 16.1 ± 0.3 years; height: 178.3 ± 5.4 cm; weight: 70.1 ± 6.2 kg) from the professional academy of a Spanish first division club participated in this study. Goalkeepers also participated in the TGs but were excluded from the data analysis. Players participated in five training sessions (80–120 min duration) and one competitive match (normally on Sunday) per week. Although the data were the result of the daily workload monitoring during the training of the team, all players were informed of the aims, requirements, benefits and risks of the study, and provided written and informed consent to participate. The research procedure was approved by the research ethics committee of the local university and was conducted in accordance with the Declaration of Helsinki.

### Procedures

**FIG. 1:**
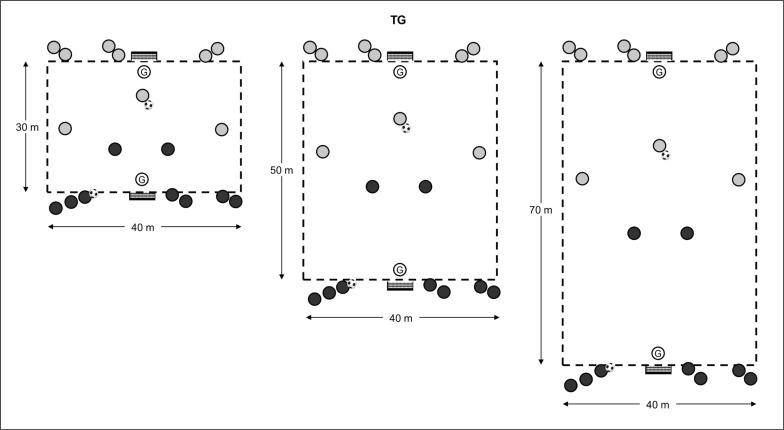
Graphical representation of TGs. TG = transition games; numbers 1 to 8 represent the evolution of all moments of each TG.

**FIG. 2:**
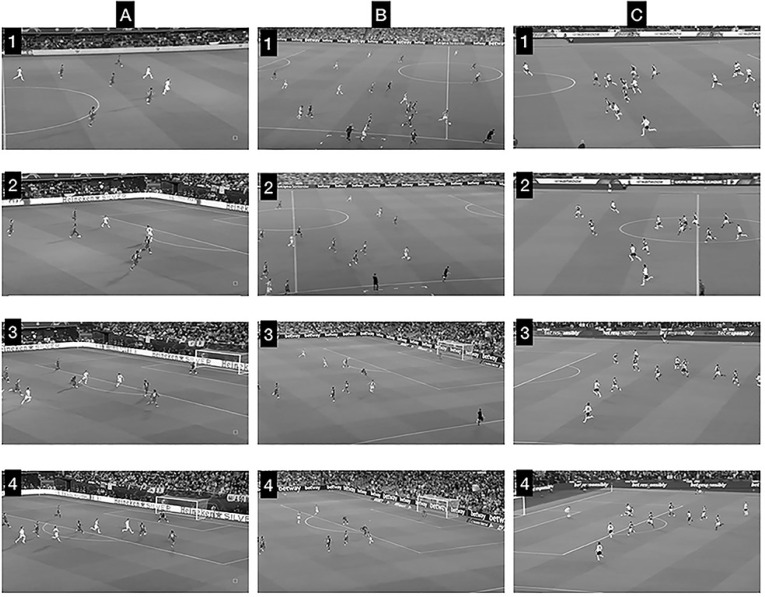
Examples of the three TGs during official matches. A = counter-attacks near to the opposite goal; B = counter-attack in the field, C = counter-attacks near to own goal. Numbers 1 = initial stage of the TG; 2 and 3 = middle stage of the TG; 4 = final stage of the TG.

Data were collected during the 2021–2022 season. External and internal training loads in the same TGs with three different pitch dimensions were monitored, as shown in Figure 1. TGs were performed in random order, being chosen by two professional coaches with experience in analysing different match situations: counter-attacks near to the opposite goal; counter-attacks in the field; and counter-attacks near to the own goal (representative images of each format during official matches are provided in Figure 2). For the TGs, players were divided into balanced teams according to technical and tactical level, competitive experience, player positions and the subjective evaluation of the coaches. Similarly to previous published work [13], two teams of 9 players operated in groups of 3 players trying to score a goal in a 3 vs 2 counter-attack where 3 players performed an offensive role and 2 were in a defensive role. After the first move, regardless of whether it was a goal or a ball loss, the defensive group rested and another three players from the same team attacked, causing a defensive transition in the opponent group. This action was repeated alternately between teams. Two consecutive actions were performed because it has been demonstrated that in official matches the mean and peak duration of transitions were around 10 s and 22–27 s respectively [9]. To avoid any disruption of play, coaches were prepared to introduce balls as needed, and they verbally encouraged the players to maintain a high work rate during the TG bouts. Several reasons justify the 3vs2 formats used. On one hand, investigations carried out on the match analysis in football have demonstrated that situations of numerical inequality often occur in competitive matches [16, 17]. On the other hand, some authors have suggested that less tactical complexity (few players) can facilitate adequate learning [18]. Finally, as can be seen in Figure 2, it is common in professional soccer. Similarly, the size of the TGs (relative area per player of 240 m^2^, 400 m^2^ and 560 m^2^ in the TG_30_, TG_50_ and TG_70_ respectively) were chosen following the literature, where it has been stated that it is necessary to have a relative area per player of ~288 m^2^ [4] or even greater, of 300 m^2^ [19], to obtain the maximal running requirements during a drill-based game. Following a standardised 20 min warm-up, a 20-min transition game was performed, distributed as follows: 2 min explanation, 12 min transition game using an intermittent format of 3 × 4 min bouts and 2 min of passive recovery between bouts. Different versions of the transition game were evaluated on Wednesday (match day -4 and after day-off). The soccer players in this study were well familiarized with these games because they were repeated several times during the season, being a frequent drill used by all the academy coaches. For the analysis, the transition games were performed three times.

### Measures

External and internal loads were monitored using a GPS system (Kinexon GNSS, Precision Technologies, Munich, Germany). Total distance covered (DC), number of accelerations and decelerations above 1.0 m · s^−2^ and 2.5 m · s^−2^ (Acc > 1.0 m · s^−2^, Acc > 2.5 m · s^−2^, Dec > 1.0 m · s^−2^ and Dec > 2.5 m · s^−2^), rate of perceived exertion (RPE), maximal heart rate (HR_max_) and time above 90% of maximal heart rate (HR_> 90_) were recorded to quantify the external and internal load. External load was evaluated through the high-speed profile of the players, using the variables distance covered between 18.0 and 20.9 km · h^−1^ (DC 18–20.9 km · h^−1^); distance covered between 21.0 and 23.9 km · h^−1^ (DC 21–23.9 km · h^−1^); distance covered at above 24.0 km · h^−1^ (DC > 24 km · h^−1^); peak speed; and sprint profile (duration, distance and maximal speed). Most of these variables have been used previously in the literature [13].

### Statistical analysis

Data are presented as mean ± standard deviation (SD). All variables presented a normal distribution (Shapiro-Wilk test). Differences between dependent internal and external loads in each transition game were determined using a repeated-measures analysis of variance. Bonferroni post-hoc tests were used to identify significant differences between parameters in each format. The level of statistical significance was set at *p* ≤ 0.05. All statistical analyses were performed using SPSS (version 19, SPSS Inc., Chicago, IL, USA). The standardized differences of effect size (ES, 95% confidence interval [95%CI]) in the selected variables were calculated. Threshold values for assessing magnitudes of the ES (changes as a fraction or multiple of baseline standard deviation) were > 0.20, 0.20, 0.60, 1.2 and 2.0 for trivial, small, moderate, large and very large, respectively [20].

## RESULTS

**FIG. 3:**
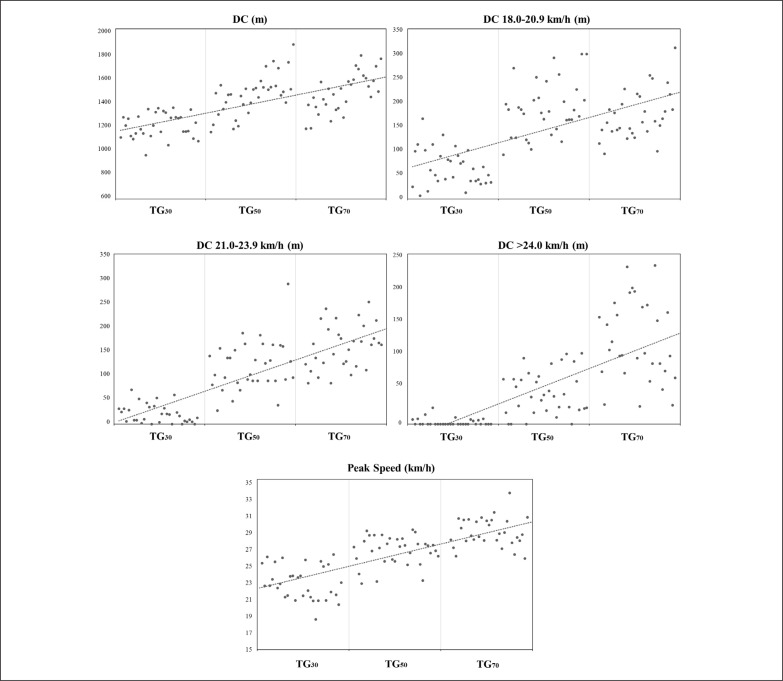
External load: distance covered and speed. TG = transition games; DC = total distance covered; max = maximal.

**FIG. 4:**
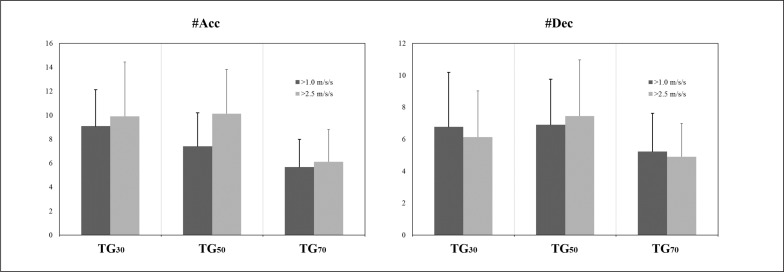
External load: accelerations and decelerations. TG = transition games; #Acc = number of accelerations; #Dec = number of decelerations

**FIG. 5:**
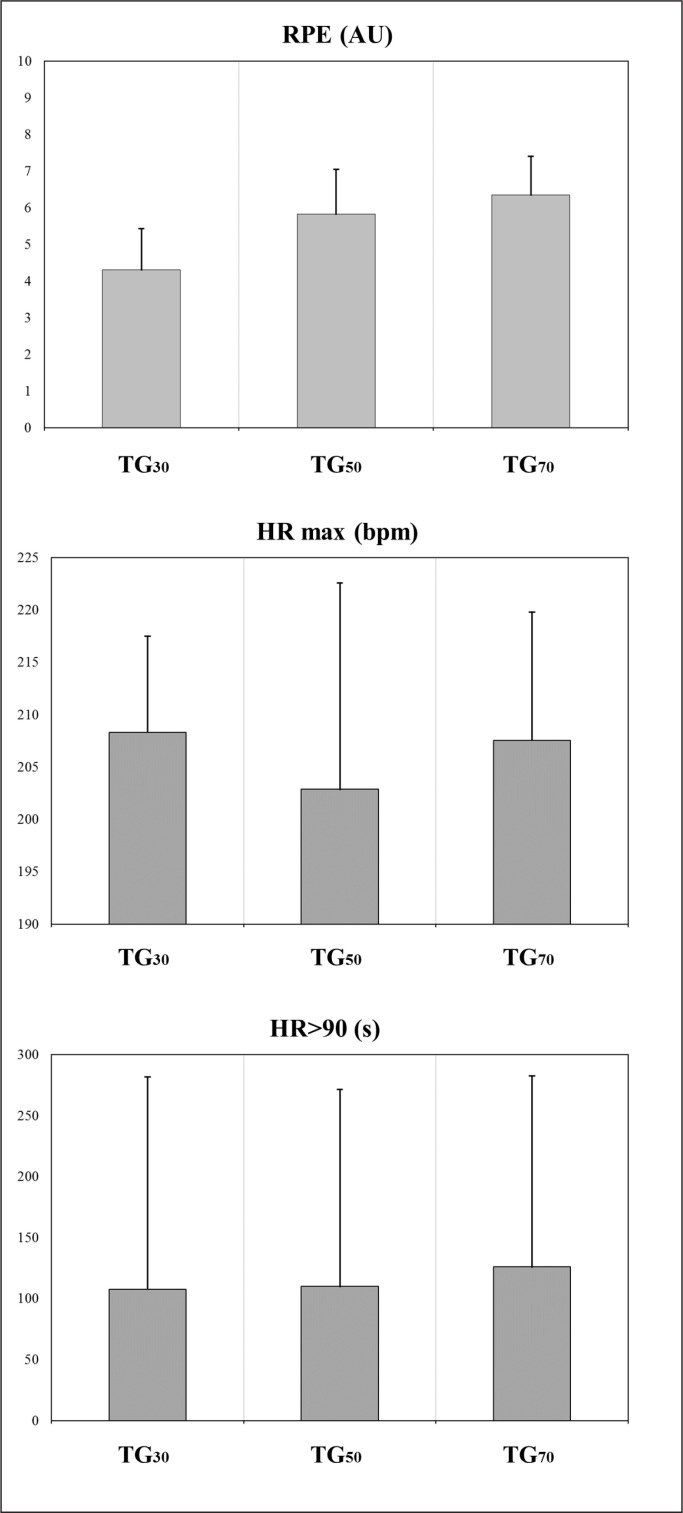
Internal load. RPE = rate of perceived exertion; AU = arbitrary units; HR = heart rate; max = maximal; bpm = beats per minute; HR_> 90_ = time above 90% of maximal heart rate.

**FIG. 6:**
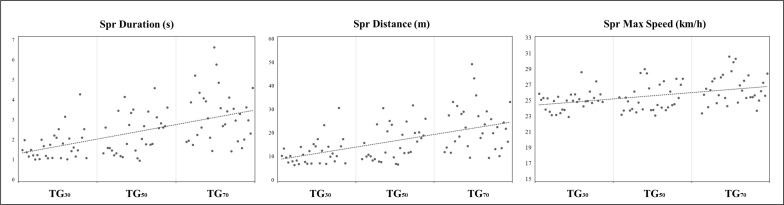
Sprint profile TG = transition games; Spr = sprint.

Descriptive statistics for the DC, accelerations-decelerations and internal load metrics measured during the three TGs are presented in Table 1, and Figures 3, 4 and 5. The comparisons of the high speed profile variables are presented in Table 2 and Figures 3 and 6. The data analyses showed that TG_30_ reached significantly lower DC, DC 18–20.9 km · h^−1^, DC 21–23.9 km · h^−1^, DC > 24 km · h^−1^, peak speed, sprint distance and RPE than TG_50_ and TG_70_ (p < 0.01 for all comparisons except RPE between TG_30_ and TG_50_ where p < 0.05), and lower sprint duration and maximal speed sprint than TG_70_ (p < 0.01). TG_30_ and TG_50_ achieved significantly higher Acc > 1.0 and > 2.5 m · s^−2^ respectively than TG_70_ (p < 0.05). TG_70_ achieved significantly greater DC 21–23.9 km · h^−1^, DC > 24 km · h^−1^, peak speed, sprint distance and maximal speed sprint than TG_50_ (p < 0.01).

**TABLE 1:** Comparison of external and internal load of TG analysed.

	Mean ± SD			p value			F	ES ± SD		
	TG_30_	TG_50_	TG_70_	TG_30_ vs. TG_50_	TG_30_ vs. TG_70_	TG_50_ vs. TG_70_		TG_30_ vs. TG_50_	TG_30_ vs. TG_70_	TG_50_ vs. TG_70_
DC (m)	1203.9 ± 100.9	1457.8 ± 164.4	1475.9 ± 160.2	0.000	0.000	0.150	35.2	1.11 ± 0.24	1.22 ± 0.20	0.17 ± 0.20
#Acc > 1.0 m · s^−2^	9.1 ± 3.0	7.4 ± 2.8	5.7 ± 2.3	0.105	0.017	0.495	12.3	-0.46 ± 0.47	-0.81 ± 0.51	-0.13 ± 0.34
#Acc > 2.5 m · s^−2^	9.9 ± 4.5	10.1 ± 3.7	6.1 ± 2.7	0.619	0.097	0.023	11.7	0.25 ± 0.88	-0.54 ± 0.53	-0.61 ± 0.42
#Dec > 1.0 m · s^−2^	6.8 ± 3.4	6.9 ± 2.8	5.2 ± 2.4	0.740	0.134	0.251	3.3	-0.11 ± 0.58	-0.49 ± 0.55	-0.28 ± 0.42
#Dec > 2.5 m · s^−2^	6.1 ± 2.9	7.4 ± 3.5	4.9 ± 2.1	0.425	0.970	0.604	6.1	-0.24 ± 0.53	0.01 ± 0.53	0.16 ± 0.54
HR max	208.3 ± 9.2	202.9 ± 19.7	207.5 ± 12.3	0.385	0.640	0.627	1.2	0.21 ± 0.41	0.08 ± 0.29	-0.11 ± 0.39
HR_> 90_ (s)	107.8 ± 174.1	109.9 ± 161.4	125.9 ± 156.7	0.686	0.795	0.783	0.5	-0.10 ± 0.45	0.09 ± 0.61	-0.09 ± 0.60
RPE (AU)	4.3 ± 1.1	5.8 ± 1.2	6.4 ± 1.1	0.000	0.048	0.575	27.7	1.07 ± 0.45	0.74 ± 0.59	0.14 ± 0.40

SD = standard deviation; ES = Effect Size; TG = Transition games; DC = total distance covered; #Acc = number of accelerations; #Dec = number of decelerations; HR = Heart Rate; max = maximal; HR_> 90_ = time above 90% of maximal heart rate; RPE = rate of perceived exertion.

**TABLE 2:** Comparison of high speed profile of TG analysed.

	Mean ± SD			p value			F	ES ± SD		
	TG_30_	TG_50_	TG_70_	TG_30_ vs. TG_50_	TG_30_ vs. TG_70_	TG_50_ vs. TG_70_		TG_30_ vs. TG_50_	TG_30_ vs. TG_70_	TG_50_ vs. TG_70_
DC 18–20.9 km · h^−1^ (m)	65.5 ± 37.6	185.5 ± 55.6	172.7 ± 49.3	0.000	0.000	0.680	60.4	1.98 ± 0.34	1.88 ± 0.42	-0.05 ± 0.19
DC 21–23.9 km · h^−1^ (m)	21.4 ± 19.3	119.6 ± 50.7	159.1 ± 46.0	0.000	0.000	0.001	93.7	2.31 ± 0.39	2.73 ± 0.44	0.48 ± 0.22
DC > 24 km · h^−1^ (m)	2.8 ± 5.4	46.0 ± 32.1	121.3 ± 58.9	0.000	0.000	0.000	76.6	1.97 ± 0.49	2.66 ± 0.46	0.97 ± 0.32
Max Speed (km · h^−1^)	22.9 ± 2.0	26.6 ± 1.7	28.8 ± 1.7	0.000	0.000	0.000	88.2	1.54 ± 0.28	2.32 ± 0.30	0.78 ± 0.26
Spr Duration (s)	1.7 ± 0.7	2.2 ± 0.9	3.3 ± 1.6	0.147	0.000	0.057	40.9	0.31 ± 0.35	1.06 ± 0.31	0.62 ± 0.52
Spr Distance (m)	11.8 ± 5.3	15.1 ± 6.9	23.1 ± 11.2	0.032	0.000	0.000	40.8	0.36 ± 0.27	0.93 ± 0.27	0.57 ± 0.14
Spr Max Speed (km · h^−1^)	25.0 ± 1.2	25.2 ± 1.5	26.0 ± 1.9	0.505	0.003	0.000	12.1	0.11 ± 0.27	0.64 ± 0.34	0.35 ± 0.15

SD = standard deviation; ES = Effect size; TG = transition games; DC = total distance covered; max = maximal; Spr = Sprint.

## DISCUSSION

In the present study we investigated the influence of three different pitch sizes in TGs on the external/internal load and the speed profile in young professional soccer players. To the best of our knowledge this is the first study that has analysed the effect of pitch size during TGs in locomotor intensity metrics. In general, the results show that enlarging the pitch size increased the total distance covered, the speed profile and the rate of perceived exertion of the players. It was also observed that mechanical load was practically unaffected by format size and only the number of accelerations was higher in the smallest pitch format.

In the relevant literature it has been shown that one of the main variables influencing training load during the development of variously sided games is the pitch size [21, 22]. In general, most studies have shown that enlarging the pitch size led players to cover greater total, running, and sprinting distances per minute than smaller formats with the same playing conditions [23–25]. Our results reinforce this idea, showing different load patterns in the different playing dimensions proposed. In particular, larger pitch sizes caused more high-speed running, sprint and peak speed demands during TGs. In accordance with previous published literature, these results may be due to two main factors. Firstly, there is a different relative area per player in the three different TGs proposed (TG_30_ = 240 m^2^; TG_50_ = 400 m^2^; TG_70_ = 560 m^2^), as has been demonstrated in variously sided games [19, 25, 26]. In the present study, when the relative area per player was increased from the TG_30_ format (240 m^2^) there was an increase in all the variables of the high-speed profile of the players, with the exceptions of sprint duration (between TG_30_ vs TG_50_ and TG_50_ vs TG_70_) and maximal sprint velocity (between TG_30_ vs TG_50_) (see Table 2). For some of these variables the enhancement observed was very significant, such as in DC > 24 km · h^−1^ (m) in which players covered 94% more distance in TG_50_ in comparison with TG_30_, and 62% more distance in TG_70_ than in TG_50_ (see Table 2). Further studies analysing the technical and tactical behaviours of the players during these TGs would be necessary to determine which soccer actions are performed during these displacements at higher speeds.

Secondly, the length:width ratio of each TG proposed (0.75, 1.25 and 1.75 arbitrary units for TG_30_, TG_50_ and TG_70_, respectively) could also have impacted the load. Previous studies have shown that a longer pitch provides more distance between the goalkeeper and the last defender, allowing the offensive players to exploit it by covering greater distances, more distance at higher intensities, and engaging in greater acceleration [27–29]. In line with these results, we observed that higher length:width ratios were associated with increased high-speed and very high-speed running. TGs are drills developed to simulate the counter-attacking situations that players experience in competition. These situations are characterized by the team in possession of the ball having large spaces in front of the opponent’s goal, with a low defensive opposition. This could explain why higher length:width ratios better simulate the speeds players produce in these situations in competition. However, in the present study, higher ratios were associated with higher relative areas per player. Further studies comparing TGs with same area per player and different length:width ratios are needed to elucidate the role of this variable format.

Comparison of our results with the only other work carried out on TGs [13] showed that the formats of 50 m and 70 m produced more similarities with the TG proposed previously (60 m) in terms of DC (1426.5 ± 100.7) DC 18–20.9 km · h^−1^ (201.5 ± 40.4), peak speed (27.0 ± 1.7) and RPE (6.4 ± 0.8). Likewise, it could be hypothesised that the performance for the variables DC 21–23.9 km · h^−1^ and DC > 24 km · h^−1^ would be the same, but unfortunately they uniquely registered the DC > 21 km · h^−1^ (255.9 ± 96.6), adding the distance covered > 24 km · h^−1^ to this zone, so these exercises are adequate to train high- and very-high speed running in counter-attack contexts [13]. In contrast, mechanical load variables (see Table 1) were practically unaffected by the different pitch sizes studied. Dec did not change and Acc > 1.0 and > 2.5 m · s^−2^ were only significantly less in the largest format with respect to the smallest (TG_30_ vs TG_70_) and the medium format (TG_50_ vs TG_70_) respectively. These results agree with others previously reported for small- to large-sided games in which smaller pitch dimensions were associated with greater mechanical load on the players [29–31]. Previous published work on TGs [13] noted greater Acc (> 1.0 m · s^−2^ = 24.0 ± 5.1; > 2.5 m · s^−2^ = 19.9 ± 3.6) and Dec > 1.0 and > 2.5 m · s^−2^ (> 1.0 m · s^−2^ 12.0 ± 3.2; > 2.5 m · s^−2^ = 14.3 ± 3.5) than were measured in the current investigation of TGs, indicating the need to continue examining Acc and Dec during TGs. In addition, considering that high-speed sprint training is considered a key factor for hamstring injury prevention, and considering the need to create specific collective exercises that accumulate high speed running during soccer training sessions [32, 33], TGs could also be used as collective preventive workouts. TG_50_ and TG_70_ could be considered suitable for preparing young soccer players for elite teams because both sizes showed similar values for sprint duration, sprint distance, and mean and maximal speed sprint to those found in high-intensity actions of professional players during regular matches [34] Another important aspect of this work is that there were no differences in the HR_max_ and HR_> 90_ between TGs, so the three formats could be considered suitable for eliciting important cardiopulmonary adaptations, as suggested by Buchheit and Laursen [35].

Although this study presents novel results from commonly used training drills, some methodological limitations should be considered. Firstly, a relatively small number of players were included for analysis and they came from only one club. However, taking into account the limited sample of professional soccer players at an elite academy, the data obtained may be useful to help coaches and sport scientists to understand the load orientation of TGs depending on the selected pitch size. Also, a specific format of TGs (3vs2) was studied, and although this format is very frequent in soccer, other TG compositions should be studied and included in future research, in order to gain a better grasp of the features of TGs. Future studies should analyse TGs performed with a larger sample involving soccer players from different professional academies executing the same task. Likewise, it appears necessary to monitor the training load of TGs carried out with different compositions, for example, 1vs1 and 2vs1.

## CONCLUSIONS

The present study shows the internal and external responses of the soccer players to different modifications of the pitch size during the TGs. The results of the present investigation reflect that the total distance covered, the speed requirements and the rate of perceived exertion of the players increase as the pitch size is increased, while the only variable that is greater in the smaller pitches is the number of accelerations.

### Practical applications

These results could help the planning process of exercise design in soccer. Considering that coaches periodize the highest weekly training load in the middle of the week [5, 36], TG_70_ and TG_50_ should be used to overload variables related to high speeds and sprints in this phase of the microcycle, for example, four or three days before the match. Similarly, technical staff could use TG_30_ during the latter part of the week (two days before the match) due to the lower load requirements. In this sense, soccer teams could train a specific counter-attack drill any day of the week depending on the desired training load or the days remaining before the match, this last aspect being especially important during congested periods.
